# Endoscopically Assisted Resection of a Rare Mass: Intra-Articular Osteochondroma of Shoulder Originated from Scapula

**DOI:** 10.1155/2016/7684807

**Published:** 2016-01-26

**Authors:** Baran Sarikaya, Fatih Suluova, Baki Volkan Cetin, Zeynep Bekin Sarikaya

**Affiliations:** ^1^Department of Orthopedics and Traumatology, Harran University Faculty of Medicine, Hamidiye Mahallesi Necmettin Cevheri Caddesi, Yenisehir, Haliliye, 63050 Sanliurfa, Turkey; ^2^Department of Orthopedics and Traumatology, Karatas Hospital, Izmir, Turkey; ^3^Department of Radiology, Balikligol State Hospital, Sanliurfa, Turkey

## Abstract

Osteochondromas are the most common benign bone tumors which are mostly seen in the metaphysis of distal femur, proximal tibia, and proximal humerus. As arising from flat bones such as scapula is a rare case, intra-articular osteochondroma is also rare. When the literature is searched it appeared that the scapula and shoulder joint are an uncommon site for osteochondroma. We present a case in which a patient had an osteochondroma placed in shoulder joint and originated from scapula which is a rare situation determined in the literature.

## 1. Introduction

Osteochondroma is the most common benign bone tumor [1, 2] and it is also known as exostosis. It is believed to arise from aberrant cartilage (perichondrial ring) on the bone surface. Tumor consists of a bony mass and a cartilage cap occurred due to persistent enchondral ossification. Osteochondroma may have a thin stalk (pediculate) or be broad based (sessile) [3–5]. Osteochondroma growth parallels the growth of the patient overall and most of the cases diagnosed in this period. Growth of the tumor ceases or slows considerably when skeletal maturity is reached [6]. Osteochondroma occurs frequently as a solitary osteocartilaginous exostosis and rarely as hereditary multiple lesions (Hereditary Multiple Exostoses, HME) [7, 8]. Malignant transformation of osteochondroma is the main concern and its incidence in solitary type is 1% and 3%–5% of patients with HME. Malignant change is characterized by sudden growth in the size of tumor after skeletal maturity. Thickness of the cartilaginous cap of osteochondroma is another suspicious criterion for malignant transformation [3, 6, 9]. Osteochondroma has usually no symptom and diagnosis is made incidentally, based on radiographs. Fracture of the stalk due to trauma is rare. These lesions resulting in symptomatic complaints include sequelae of surrounding soft tissue impingement including bursa formation and vessel and nerve impingement. Surgical removal is only indicated for significant clinical symptoms.

We present a case in which a patient had pain and swelling of the shoulder due to osteochondroma placed in uncommon site in shoulder joint.

## 2. Case Report

**Figure 1:**
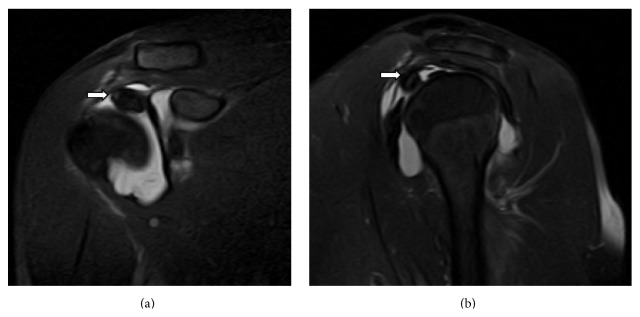
MR imaging of osteochondroma: coronal (a) and sagittal (b) T2 TSE SPIR images.

A 39-year-old man, with no history of trauma, presented to our hospital with pain and swelling in the right shoulder. Pain and limitation of movement had been increasing over a year. Patient, firstly, had been examined in physical therapy department and performed range-of-motion exercises. During this treatment, swelling was added to his complaints. Since pain and swelling developed gradually patient was directed to our department. On the first examination we detected large swelling on the right shoulder and external rotation, flexion, and extension and in particular internal rotation of shoulder was limited. X-ray examination showed that no abnormal finding was noted. MRI revealed the presence of a 2 cm bony mass adjacent to subscapular tendon and close to the glenoid with the typical features of an osteochondroma and extensive effusion in the shoulder joint (Figure 1).

**Figure 2:**
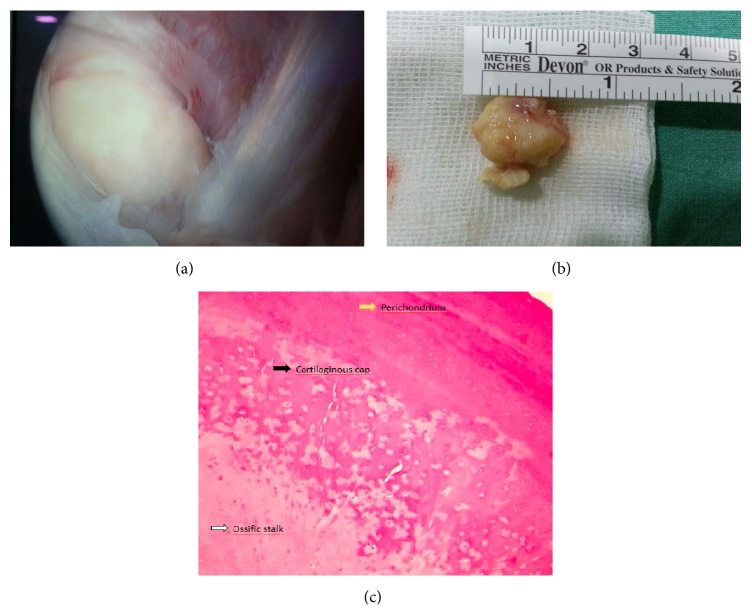
Before excision (a), after excision (b), and microscopical view (c) of osteochondroma.

Endoscopically assisted surgery was planned. Under general anesthesia, the patient was placed in lateral decubital position. Endoscopic intervention was implemented through the proper portals. First extensive effusion was identified related to synovitis. A 2 cm bony mass was visualized superior to subscapular muscle tendon and close to anterosuperior site of glenoid. Consequently, the tumor was excised through its base by the way of anterior portal (Figure 2). There was no evidence of degeneration or rotator cuff rupture.

At clinical examination 1 week postoperatively, the patient had dramatic recovery and regained near-full motion of the shoulder, which was almost pain-free.

## 3. Discussion

Generally osteochondroma has no symptom, and diagnosis is made incidentally based on posttrauma X-ray films as a bony mass without pain. Osteochondromas may result in symptomatic complaints that include pain due to adjacent soft tissue impingement including bursa formation, vessel and nerve impingement, and painless cosmetic deformity related to the slowly enlarging exophytic mass (pseudowinging scapula). When the tumor is placed by tendon and bursa formation or as in our case intra-articularly it can become symptomatic. Surgical decision can be made in such circumstances. Aside from pain and cosmetic issues, surgical removal may also be indicated due to malignant transformation which is determined to be 1-2% [10]. It has been shown that this incidence can be increased when cartilaginous cap is thicker than 2 cm [6, 11]. In addition malignant transformation is seen in 5–25% with MHE [10].

In our case patient had performed range-of-motion exercises but during treatment had no benefit; besides, pain and swelling were getting worse. Osteochondromas may become symptomatic due to pressure on the surrounding soft tissue such as tendon, bursa, vessel, nerve, and the other anatomic structures. Thus, in the present case, osteochondroma caused pain which was placed intra-articularly and near the subscapular muscle tendon. Joint motion exercises increased irritation of the tendon and synovitis after intra-articular irritation statement was also exacerbating the existing pain clinics. Surgical excision as a result of all these is inevitable for our patients. The patient showed dramatic improvement after the surgery was performed for the mass causing the irritation.

In the literature, osteochondral lesions were found to be the most common in metaphyseal site of long bone close to physis, distal femur, proximal tibia, and proximal humerus [4, 12]. Although there does not exist a typical place for osteochondroma, as in our case presentation, it may also be seen as ankle, elbow, and shoulder joints [1, 13–15]. Osteochondromas can be symptomatic after years from completion of the skeletal system development [16]. This condition can be supposed by the lack of symptoms in our 39-year-old patient until last year. On the other hand scapula, due to a flat bone osteochondroma, is also a rare anatomical localization. In the literature, the incidence of osteochondromatosis in the scapula was found between 3 and 4.6% and osteochondromatosis constitutes 49% of benign tumors of scapula [17, 18]. Settlement of the scapula is so often ventral side [4]. Pain due to mass effect, snapping scapula, and pseudowinging scapula can cause conditions such as restriction of movement of the shoulder joint [19, 20]. Analyzing the literature, our case is a rare condition, due to location of osteochondroma both on the scapula and on intra-articular area.
